# Comparative evaluation of physical and mechanical properties of ion-releasing and non-ion-releasing bulk-fill restorative composites

**DOI:** 10.12669/pjms.42.4.13960

**Published:** 2026-04

**Authors:** Majed M. Alsarani, Manal Al-Mutairi, Aftab Ahmed Khan

**Affiliations:** 1 Majed M. Alsarani Dental Health Department, College of Applied Medical Sciences, King Saud University, Riyadh, Saudi Arabia; 2 Manal Al-Mutairi Pediatric Dentistry and Orthodontics Department, College of Dentistry, King Saud University, Riyadh, Saudi Arabia; 3 Aftab Ahmed Khan Dental Health Department, College of Applied Medical Sciences, King Saud University, Riyadh, Saudi Arabia

**Keywords:** Bulk-fill composites, Bioactive restorative materials, Ion-releasing, Mechanical properties, Physical properties

## Abstract

**Objective:**

This laboratory study aimed to compare the performance of non-ion and ion-releasing restorative composites.

**Methodology:**

This *in vitro* experimental study was conducted at King Saud University from March 2025 to June 2025. The study groups included four bulk-fill restorative composites, consisting of two conventional non-ion-releasing formulations—Filtek Bulk and Tetric EvoCeram Bulk Fill—and two ion-releasing formulations—Beautifil Bulk Restorative and ACTIVA BioACTIVE Bulk. A total of 25 samples were prepared per group: five disc-shaped (n = 5/group) for depth of cure, ten disc-shaped (n = 10/group) for water sorption and solubility, and ten bar-shaped (n = 10/group) for bending strength testing. The one-way analysis of variance followed by Tukey’s *post hoc* tests for multiple comparisons was conducted with a significance level set at *P* = 0.05.

**Results:**

Filtek Bulk demonstrated the highest depth of cure (4.34 ± 0.29 mm) and bending strength (123.34 ± 9.05 MPa), succeeded by Tetric EvoCeram Bulk Fill (3.81 ± 0.17 mm, 115.78 ± 10.98 MPa). Additionally, Filtek Bulk and Tetric EvoCeram Bulk Fill exhibited significantly lower water sorption (0.49 ± 0.13% and 0.79 ± 0.10%, respectively) and solubility (0.01 ± 0.00% and 0.04 ± 0.00%, respectively) compared to ion-releasing composites, Beautifil Bulk Restorative and ACTIVA BioACTIVE Bulk (1.26 ± 0.19% and 1.42 ± 0.20%; 0.10 ± 0.01% and 0.14 ± 0.01%, respectively) (P < 0.05).

**Conclusion:**

While ion-releasing composites offer the benefit of reducing secondary caries risk and remineralization of the tooth, their physical and mechanical properties may not be on par with those of conventional non-ion-releasing restorative composites, potentially affecting their long-term performance in deep restorations. Therefore, the clinicians are advised to exercise caution in using these restorative composites.

## INTRODUCTION

With the advent of bulk fill dental composites in markets, the restorative procedure has become faster, with improved clinical efficiency and patient comfort.^1^ Among the several advantages, the ability to cure in 4-5 mm increments, reducing the need for multiple layers and saving clinical chair time are worth mentioning.^2^ Compared to earlier generations of composite materials, bulk-fill composites have been modified to incorporate a lower filler volume load and larger filler particles with reduced surface areas. These modifications facilitate greater depth of cure with reduced shrinkage and shrinkage stress.^3^

Clinicians must comprehend the maximum permissible thickness for each layer of restorative composite components. Applying layers thicker than is advised might weaken polymerization at deeper levels, which could lead to decreased biocompatibility, physical and mechanical performance.^4^,^5^ While several mechanical properties affect the quality and performance of restorative materials, bending strength is especially critical as it evaluates the material’s ability to withstand compressive, tensile, and shear forces, which closely mimic the functional loads encountered in the oral cavity.^6^

Likewise, the significance of water sorption and solubility is paramount, since excessive water sorption may lead to detrimental consequences such as swelling, oxidation, hydrolysis, softening, and plasticization.^7^ Ideally, restorative materials should demonstrate low permeability and high resistance to water sorption and solubility to maintain their structural integrity and long-term durability.^8^,^9^

With the marketing of newly ion-releasing bulk-fill dental composites, it is imperative to conduct laboratory testing to evaluate their fundamental physical and mechanical properties. Such assessments are crucial not only for understanding material performance but also for providing evidence-based guidance to dental practitioners on their proper usage to achieve optimal clinical outcomes and ensure long term durability. The need for more research is highlighted by the fact that it is yet unknown if the depth of cure, mechanical and physical properties of these ion-releasing restorative composites are comparable to those of conventional bulk-fill composites. Therefore, this study aimed to assess the depth of cure, bending strength, water sorption and solubility of two ion-releasing restorative composites and compared their performance with widely used non-ion-releasing bulk-fill restorative composites. The scraping technique was used to evaluate the depth of cure.^10^ The hypothesis was that the physical and mechanical properties of ion-releasing bulk-fill restorative composites would be at par with those of conventional non-ion-releasing composites.

## METHODOLOGY

This *in vitro* experimental laboratory study was performed at the Dental Health Department, College of Applied Medical Sciences, King Saud University, Riyadh, Saudi Arabia, between March and June 2025.

### Ethical Approval:

The Institutional Review Board granted an exemption for this research (25/0009/IRB; dated January 7, 2025) as it did not involve any human or animal subjects.

Four commercially available bulk-fill restorative composites were selected, comprising two conventional non-ion-releasing: Filtek Bulk (3M ESPE, USA) and Tetric EvoCeram Bulk Fill (Ivoclar Vivadent, Liechtenstein) and two ion-releasing formulations: Beautifil Bulk Restorative (Shofu, Japan) and ACTIVA BioACTIVE Bulk (Pulpdent Corporation, USA). Fifteen disc-shaped samples, each measuring 6-mm in diameter and 4-mm in thickness, were prepared for depth of cure, water solubility and sorption tests from each study group using a silicone mold. A composite material was filled inside the mold and covered with a glass slide. The sample was cured for 40 seconds from the top using a handheld LED curing device (Freelight 2, 3M ESPE, Seefeld, Germany) with a 10 mm tip diameter. The light-curing tip was positioned in direct contact with the glass slide surface during polymerization to ensure maximum light intensity and uniform curing. The light irradiance was measured with a calibrated radiometer and recorded as approximately 1000 mW/cm². Similarly, ten bar-shaped samples from each group, measuring 30 mm × 2 mm × 2 mm, were fabricated for the bending strength test. The sample was light-cured in increments for 40 seconds each to ensure homogeneous curing throughout the length of the sample. During polymerization, the device was positioned in close contact with the glass slide with a gentle hand force to minimize voids. To ensure complete polymerization, the samples were taken out, allowed to air dry, and then kept in distilled water for 24 hours before testing.

The testing (depth of cure, bending strength, water solubility and sorption) was conducted under controlled laboratory conditions at 23 ± 2°C and 50 ± 5% relative humidity, following ISO 4049 specifications for dental resin-based materials.

### Depth of cure:

For the evaluation of depth of cure, a mold (6-mm in diameter and-4 mm deep) was used, and the study materials were condensed and light-cured for 40 seconds as described above. Next, the samples (n=5/group) were removed from the mold, and the uncured material was scraped away with a plastic spatula. The depth of the remaining cured restorative composite was measured with a digital micrometer at three points, and the average value of the sample was obtained.

### Bending strength:

A bending test was conducted with a total of 10 samples (n = 10/group). A sample was placed horizontally on two parallel supports with a span length of 20 mm on a universal testing machine (Model no. 3369 Instron, Canton, MA, USA). A load cell with a capacity of 5 kN was used, and the cross-head speed was set at 1.0 mm/min. The maximum load at fracture (in megapascal, MPa) was automatically recorded using the proprietary software (Bluehill ver. 2.4).

### Water solubility (Wsol) and sorption (Wsp):

The samples (6-mm in diameter and 4-mm in thickness) from each group (n=10/group) were originally dried using silica gel for two hours, followed by incubation at 37 °C for 24 hours to attain a stable weight. Their original weight (m1) was recorded with a high-precision electronic scale (Precisa, EP 320A; Dietikon, Switzerland). After this, the samples were stored in water for seven days to determine their wet weight (m2). Following a 24-hour dehydration period at 37 °C in an incubator, the final dry weight (m3) was determined. Percentages of Wsol and Wsp were calculated with the following equations:

Wsp = 100 × (m2 − m1)/m1 (1)

Wsol = 100 × (m1 − m3)/m1 (2)

### Statistical analysis:

The collected data were analysed using both descriptive and inferential statistical methods. Descriptive statistics involved calculating the mean and standard deviation values whereas inferential statistics involved a one-way analysis of variance (ANOVA), followed by Tukey’s HSD *post hoc* tests for multiple comparisons, with a significance level set at p = 0.05. All statistical evaluations were performed using SPSS software, version 23.0 (IBM, New York, USA).

## RESULTS

The depth of cure potential of the study groups is mentioned in Table-I.. The highest depth of cure (in mm) was seen in Filtek Bulk (4.34 ± 0.29 mm), which was significantly higher than the depth of cure of Tetric EvoCeram Bulk Fill (3.81 ± 0.17 mm) and ACTIVA BioACTIVE Bulk (3.79 ± 0.90 mm). Beautifil Bulk Restorative showed the depth of cure to 4.11 ± 0.21 mm. The details are in Table-I.

**Table-I:** Comparison of depth of cure (in mm) among ion-releasing and non-ion releasing dental restorative composites.

Group	Depth of cure (in mm)	P value
Filtek Bulk	4.34 ± 0.29^a,b^	0.002
Beautifil Bulk Restorative	4.11 ± 0.23	
Tetric EvoCeram Bulk Fill	3.81 ± 0.17^a^	
ACTIVA BioACTIVE Bulk	3.79 ± 0.90^b^	

***Note:*** The same superscript lowercase letters depict a statistical difference between the groups.

The bending strength values (in MPa) of the study materials is shown in Table-II. The highest bending strength was observed in Filtek Bulk (123.34 ± 9.05 MPa), which was significantly higher than Beautifil Bulk Restorative (96.21 ± 8.10 MPa) and Activa Bioactive Bulk (92.91 ± 6.85 MPa). Similarly, Tetric EvoCeram Bulk Fill (115.78 ± 10.98) showed significant differences with Beautifil Bulk Restorative and ACTIVA BioACTIVE Bulk.

**Table-II:** Comparison of bending strength (in MPa) among ion-releasing and non-ion releasing dental restorative composites.

Group	Bending strength (in MPa)	P value
Filtek Bulk	123.34 ± 9.05^a,b^	<.001
Beautifil Bulk Restorative	96.21 ± 8.10^a,c^	
Tetric EvoCeram Bulk Fill	115.78 ± 10.98^c,d^	
ACTIVA BioACTIVE Bulk	92.91 ± 6.85^b,d^	

***Note:*** See Table-I.

The water sorption data, expressed as percentages, for the evaluated study groups are4 displayed in Table-III.. The non-ion-releasing restorative composites exhibited significantly lower water sorption compared to the ion-releasing restorative composites (P < 0.05). Among the tested materials, Filtek Bulk demonstrated the lowest water sorption potential (0.49 ± 0.13%), whereas ACTIVA BioACTIVE Bulk exhibited the highest water sorption potential (1.42 ± 0.20%).

**Table-III:** Comparison of water sorption (in %) among ion-releasing and non-ion releasing dental restorative composites.

Group	Water sorption (in %)	P value
Filtek Bulk	0.49 ± 0.13^a,b,c^	<.001
Beautifil Bulk Restorative	1.26 ± 0.19^a,d^	
Tetric EvoCeram Bulk Fill	0.79 ± 0.10^b,d,e^	
ACTIVA BioACTIVE Bulk	1.42 ± 0.20^c,e^	

***Note:*** See Table-I.

A comparison of the water solubility values among the tested restorative composites is presented in Table-IV.. Filtek Bulk demonstrated the lowest water solubility (0.02 ± 0.00%), whereas ACTIVA BioACTIVE Bulk exhibited the highest water solubility potential (0.17 ± 0.01%). Statistically significant differences were observed among all groups (P < 0.05).

**Table-IV:** Comparison of water solubility (in %) among ion-releasing and non-ion releasing dental restorative composites.

Group	Water solubility (in %)	P value
Filtek Bulk	0.01 ± 0.00^a,b,c^	<.001
Beautifil Bulk Restorative	0.10 ± 0.01^a,d,e^	
Tetric EvoCeram Bulk Fill	0.04 ± 0.00^b,d,f^	
ACTIVA BioACTIVE Bulk	0.14 ± 0.01^c,e,f^	

***Note:*** See Table-I.

## DISCUSSION

The findings revealed significant differences in the physical and mechanical properties between ion-releasing and non-ion-releasing restorative composites. Therefore, the hypothesis is rejected. The curing depth of any restorative composite is crucial in evaluating the mechanical properties, clinical life and performance.^11^ In this study, we used an LED curing device with an irradiance ≈ of 1000 mW/cm². A light irradiance of ≈ 1000 mW/cm² is considered sufficient for polymerization. We believe that factors such as high translucency, reduced scattering due to uniformly dispersed fillers, and optimized filler size^12^ might have helped raise the degree of conversion in Filtek Bulk and Beautifil Bulk Restorative.

A comparatively lower depth of cure in Tetric EvoCeram Bulk Fill could be attributed to the opacifiers incorporated in its composition, which may reduce light transmission.^13^ Although the manufacturer includes an initiator booster (Ivocerin) intended to enhance polymerization at greater depths, this enhancement may not fully compensate for the limited light penetration in deeper layers.^14^ Whereas, the dual-curing mechanism of ACTIVA BioACTIVE Bulk might lead to uneven polymerization during light exposure,^15^ especially in deeper layers. Compared to previous studies, our findings contrast with those of Alrahlah et al., who reported a higher depth of cure for Tetric EvoCeram Bulk Fill compared with Filtek Bulk Fill.^16^ Similarly, Parasher et al. reported a higher degree of cure for Tetric EvoCeram Bulk than for Beautifil Bulk Restorative.^17^ Clinicians are therefore advised to exercise caution when using these materials in deep cavities, as certain formulations demonstrate inadequate curing beyond 4 mm.

The higher bending strength of Filtek Bulk restorative composite could be due to the optimal filler ratio in the matrix and a chemical bond between them.^18^ When the optimal ratio of filler is compromised, the mechanical properties of the restorative composite are affected.^19^ The amount and type of the fillers as a reinforcing agent increase/decrease the bending properties of a restorative composite.^20^ The highest bending strength observed in Filtek Bulk and comparable bending strength in Tetric EvoCeram Bulk Fill could be due to their high filler content, enhanced resilience, and capacity to endure greater stress before fracturing.^21^ Whereas a moderate bending strength in Beautifil Bulk Restorative could be due to slightly lower filler loading (~70 wt%) and polymerization efficiency. While the dual-curing mechanism of ACTIVA BioACTIVE Bulk may lead to uneven polymerization and, consequently, affect its mechanical properties.^15^ A previous study also reported that the optimal filler weight ratio is a key determinant of the bending properties of composite materials.^22^

The water sorption values correlate well with the depth of cure and bending strength data. The lowest water sorption in Filtek Bulk is likely due to its high filler loading (~76.5 wt%), reduced resin matrix volume^8^, and a densely cross-linked polymer network, which collectively restrict water diffusion into the material. Previous polymer-based investigation has also demonstrated that increased filler content and reduced free volume within the polymer matrix effectively limit water uptake and hydrolytic plasticization, thereby preserving mechanical stability.^23^ Besides, a cross-linked polymer network contributes to enhancing the physical properties, such as resisting solvents.^24^ The higher alkaline fillers in ACTIVA BioACTIVE Bulk are designed to release fluoride, hydroxide, and calcium ions under water immersion to facilitate its proposed remineralizing effect, which may lead to greater water sorption.^25^ Similarly, bioactive fillers in Beautifil Bulk Restorative may increase its hydrophilicity. These ion-releasing restorative composites are prone to water sorption. While intermediate water sorption values of Tetric EvoCeram Bulk Fill might suggest a balance between filler content (~77 wt%) and resin matrix composition.

Solubility of bulk-fill restorative composites in water is primarily attributed to the release of unreacted residual monomers, additives, and filler constituents.^26^ After water sorption, the dissolution and disintegration of the material into the solvent takes place. Therefore, water sorption is closely related to solubility.^25^ The water solubility data are in line with the sorption data. Release of ions creates surface voids that may contribute to higher water solubility in ion-releasing restorative composites compared to non-ion-releasing composites.

### Strength of the study:

The findings of this research suggest that the physical and mechanical properties of ion-releasing and non-ion-releasing restorative composites greatly differ in terms of depth of cure, bending strength, water sorption and solubility. This is among the few comparative studies to simultaneously evaluate depth of cure, bending strength, water sorption, and solubility in currently marketed bulk-fill composites with differing functional claims. Therefore, patient-specific needs and clinical scenarios must guide the choice of the clinicians. Patients with high caries risk and poor oral hygiene should be treated with ion-releasing restorative composites for remineralization and impeding secondary caries. Conversely, non-ion-releasing composites are more suitable for patients with good oral hygiene who primarily require high esthetics, durability, strength, and polish ability.

### Limitations:

This study too had some limitations, such as testing was performed in the absence of intraoral conditions. Secondly, the ageing effects, *e.g*., hydrolytic degradation, could not be evaluated to predict long-term durability. In addition, this study did not incorporate mechanical fatigue loading to simulate repetitive masticatory stresses in the oral environment. In future, clinical studies need to be conducted to evaluate the long-term performance of these bulk-fill restorative composites.

## CONCLUSION

From the obtained results, we conclude that the composition, filler type and ratio greatly influence the physical and mechanical properties of the tested restorative composites. Filtek Bulk and Tetric EvoCeram Bulk Fill showed better depth of cure and bending strength, along with significantly lower water sorption and solubility, indicating greater structural stability and resistance to hydrolytic degradation. In contrast, ion-releasing composites (Beautifil Bulk Restorative and ACTIVA BioACTIVE Bulk) exhibited higher water sorption and solubility. These findings confirm a clear inverse relationship between mechanical durability and bioactive functionality. Therefore, material selection must be guided by clinical demands, balancing strength and longevity against therapeutic benefits.

### Authors’ contributions:

**MMA:** Conception, literature search, and responsibility for the integrity of this study.

**MAM:** Design, Interpretation**,** analysis and critical review.

**AAK:** Methodology, data collection and data interpretation, writing manuscript, Supervision and revision.

All authors have read and approved the final version.
